# Dietary Intervention with Whey Protein Concentrate Does Not Affect Toll-like Receptor Responses and Gene Expression Patterns in Peripheral Blood Mononuclear Cells of Healthy Volunteers

**DOI:** 10.3390/nu16050592

**Published:** 2024-02-22

**Authors:** Mojtaba Porbahaie, Laurien H. Ulfman, Andrei Prodan, Malgorzata Teodorowicz, Joyce E. L. Schloesser, Huub F. J. Savelkoul, Alwine F. M. Kardinaal, R. J. Joost van Neerven

**Affiliations:** 1 Cell Biology and Immunology, Wageningen University & Research, 6708 WD Wageningen, The Netherlands; 2 FrieslandCampina, 3818 LE Amersfoort, The Netherlands; 3 NIZO Food Research, 6718 ZB Ede, The Netherlands

**Keywords:** dietary intervention, whey protein, PBMCs, gene expression, TLR response, cytokine

## Abstract

Bovine milk contains bioactive proteins, carbohydrates, and phospholipids with immunomodulatory properties impacting human immunity, potentially contributing to resistance to infections and allergies through diverse mechanisms. One such mechanism is the enhancing of the innate immune response to secondary pathogen-related stimuli, termed innate immune training. Although *in vitro* studies demonstrate that milk immunoglobulin G (IgG) can train human monocytes, evidence for *in vivo* immune training is limited. To explore the potential of bovine IgG for inducing innate immune training *in vivo*, this human study utilized an IgG-rich whey protein concentrate (WPC). Healthy male volunteers were assigned to a high dose WPC, low dose WPC, or placebo group. Blood was collected pre- and post-two weeks of WPC consumption. Peripheral blood mononuclear cells (PBMCs) were isolated and stimulated with TLR ligands, evaluating IL-6 and TNF-α production by monocytes, myeloid DCs, and plasmacytoid DCs. Additionally, RNA was isolated for differential gene expression (DGE) analysis. Results indicated that the two-week WPC intervention did not influence the *ex vivo* response of studied cells to TLR agonists. Furthermore, PBMC gene expression patterns showed no significant differences between the placebo and high dose WPC groups. The data suggests that oral WPC ingestion did not enhance immune responses in young, healthy male participants.

## 1. Introduction

Milk is a complex fluid containing hundreds of components that support healthy growth and development, including proteins, lipids, carbohydrates, and micronutrients. In addition to its nutritional values, several milk components have been shown to have immunomodulatory effects [1,2,3,4]. In their intact bioactive forms, these components influence physiological processes at multiple levels and locations and can, for example, modulate innate and adaptive immune responses, as well as microbiota composition, ultimately contributing to immune health [5,6,7,8,9,10]. In combination with the extensive consumption of bovine milk and the use of milk in early life nutrition, milk or its components are attractive candidates for nutritional intervention strategies.

Immunologically active components of bovine milk were shown to decrease infection and allergy incidence [5,6,7,8,9,10]. Milk-fat-globule membrane (MFGM) ingestion increased the resistance to diarrheagenic *E. coli* in healthy adults [11], and fortifying infant formula with bovine milk lactoferrin was shown to reduce the incidence of diarrhea and respiratory tract infections in weaned infants [9]. Moreover, consumption of bovine immunoglobulins from milk or colostrum of immunized cows decreased enterotoxigenic *E. coli* (ETEC)-induced diarrhea [12,13] as well as rotavirus infection [14,15]. In addition, infants consuming raw cow’s milk have a reduced incidence of common respiratory infections [16], whereas TGF-β and other components present in raw bovine milk contribute to establishing a regulatory environment that decreases T helper 2 (Th2) responses associated with allergic reactions [17,18].

Interestingly, milk-derived IgG has been shown to improve the responsiveness of human monocytes *in vitro* when stimulated with toll-like receptor (TLR) ligands [19]. This phenomenon is known as innate immune training or trained immunity. In this concept, monocytes are primed to have an increased response to pathogen-derived TLR ligands after primary exposure to a training-inducing component [18,20,21,22]. These changes are linked to epigenetic reprogramming of the cells at the chromatin organization level, including DNA methylation and a shift in cellular metabolism [20]. The last two studies mentioned above used raw milk, purified bIgG, and whey protein concentrate (WPC) high in bIgG as training agents *in vitro*, leading to enhanced IL-6 and TNF-α production after stimulation with the TLR ligands LPS (TLR 4) and R848 (TLR7/8) [19]. Bovine IgG is not thought to reach circulation after ingestion [23], but it may interact with extended dendrites of dendritic cells in the mucosa of the gastrointestinal tract [24] and may also interact directly with immune cells in the tonsillar crypts of Waldeyer’s ring [25]. However, it is not clear if the *in vitro* findings can be extrapolated to enhanced monocyte responses and gene expression patterns *in vivo* after nutritional intervention with food ingredients rich in bovine IgG.

A recent study was conducted on the impact of consuming a whey protein concentrate (WPC) on diarrheagenic *E. coli* infection using the same *E. coli* challenge model [26]. WPC (Vivinal MFGM) is a whey protein concentrate containing bioactive whey proteins (including high levels of bovine IgG), MFGM, and phospholipids. For the current study, blood samples were analyzed from a randomly selected subgroup of the participants of the study mentioned above. To assess if nutritional intervention with WPC can enhance immune responses *in vivo*, as previously seen for primary diarrheagenic *E. coli* infection, we examined whether dietary intervention with bovine IgG-rich WPC improves monocyte and dendritic cell (DC) response to diarrheagenic *E. coli* and other TLR stimuli *ex vivo*. In addition, we studied whether nutritional intervention with WPC influenced the gene expression patterns in peripheral blood mononuclear cells (PBMCs) of the participants by isolating RNA and performing differential gene expression (DGE) analysis.

## 2. Material and Methods

### 2.1. Study Design, Participants, and Specimens

The study protocol was approved by the Medical Ethics Committee (METC) of Brabant, Tilburg, The Netherlands (July 2019), and registered as NL66645.028.18. In addition, the study was recorded with the Netherlands Trial Register as NTR7613.

**Figure 1:**
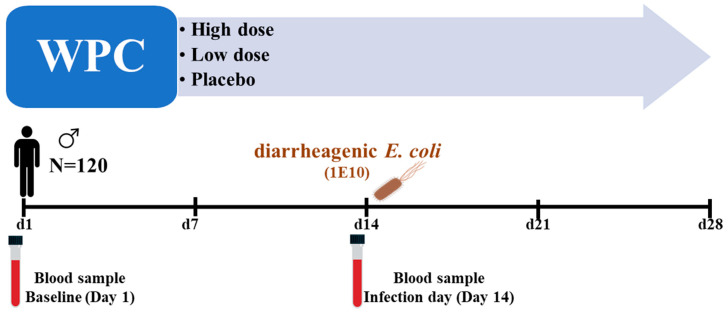
Graphical presentation of the study events: 120 healthy male volunteers were included in the main study. They were randomized into three dose groups: WPC high, WPC low, and placebo. After two weeks of product consumption, they were orally challenged with 1 × 10^10^ CFU of diarrheagenic *E. coli* strain E1392/75-2A. Blood samples were collected on day 1 (baseline) and day 14 (challenge day) from 48 randomly selected participants for the current study.

The main study enrolled 120 healthy male volunteers (age 18–55 years) who met all of the inclusion criteria and none of the exclusion criteria (Supplementary Data). Participants were randomly assigned to one of three treatment groups (*n* = 40 per group) throughout this double-blind, parallel 4-week intervention trial—control hydrolyzed whey product (placebo), high dose, or low dose of the study product, WPC. The participants were instructed to maintain their usual physical activity and food intake while limiting their calcium intake to a maximum of 500 mg/day. Participants ingested the study product twice daily for four weeks; the high dose group (23 g/serving of WPC), the low dose group (11 g/serving of WPC supplemented with 12 g/serving control whey hydrolysate), and the placebo group (23 gr/serving of control whey hydrolysate). The participants were orally challenged with 1E10 CFU of a live attenuated *E. coli* strain E1392/75-2A on day 14 of the study (Figure 1). A separate paper discusses the evaluation of clinical effects following the infectious challenge [26].

The present research examined a randomly chosen subpopulation of the main study [26]. Randomization was performed by using ResearchManager software version 5.40 (ResearchManager, Deventer, The Netherlands). Blood samples were taken from 48 participants (placebo *n* = 19, WPC low dose *n* = 10, WPC high dosage *n* = 19) at baseline (day 1) and on day 14 of the trial, shortly prior to the infectious challenge. The aim was to evaluate the *ex vivo* response of PBMCs to TLR ligands and PBMC gene expression analyses.

### 2.2. Ex Vivo Stimulation of Monocytes, mDC, and pDC by Diarrheagenic E. coli and TLR Ligands

#### 2.2.1. PBMC Isolation

On days 1 (baseline) and 14 (challenge), participants’ whole blood was taken in BD Vacutainer CPT^TM^ tubes (Becton Dickinson 362761, Franklin Lakes, NJ, USA). To isolate PBMCs, the tubes were directly centrifuged (1800× *g*, 25 min, room temperature) in a swinging bucket rotor. The upper interphase containing the buffy layer and plasma was then washed with 40 mL of warm PBS and centrifuged at 250× *g* (7 min, RT). After repeating the washing procedure twice, the cells were resuspended in 1 mL of RPMI-1640 and prepared for cell counting. Following plating (2 × 10^6^ cells/well) in 12-well plates (Costar CL3513, Sigma–Aldrich, St. Louis, MO, USA), the PBMCs were stimulated with medium (RPMI-1640) or TLR ligands: LPS (200 ng/mL—Sigma L2880, Sigma–Aldrich, St. Louis, MO, USA), flagellin (500 ng/mL—Invivogen tlrl-s) or the whole diarrheagenic *E. coli* strain E1392/75-2A (1E7 CFU/well). Brefeldin A (BFA) (Invitrogen 00-4506-51, Carlsbad, CA, USA) was used to inhibit cytokine excretion from the cells, and the plates were incubated for 3 h at 37 °C with 5% CO_2_.

#### 2.2.2. FACS Staining

Following a 3-h incubation, the cells were harvested and labeled with fluorochrome-conjugated antibodies against extracellular markers for PBMC phenotyping (Table 1. [Panel1]). The cells were incubated with the first antibody mixture diluted in FACS buffer (PBS + 5% BSA + 2 mM EDTA) for 30 min, wrapped in aluminum foil on ice (4 °C). Following that, the dead cells were stained with eFluor 520 Fixable Viability Dye (eBioscience 65-0867-14, San Diego, CA, USA).

After cell fixation and membrane permeabilization with IC fix/perm kit (Invitrogen, #88-8824-00, Carlsbad, CA, USA), the intracellular production of IL-6 and TNF-α was assessed by staining the cells with flow cytometry antibodies included in the second antibody mixture (Table 1. [Panel2]). The supplementary data contains the stepwise staining procedure. The stained samples were then measured on a Beckman Coulter Cytoflex LX, and the data were analyzed using FlowJo v10 (FlowJo LLC, Ashland, OR, USA). The supplementary data describe the gating strategy used to identify different cell types and cytokine production profiles. Paired sample *t*-test was performed to compare the baseline and day 14 responses within each group, and differences were declared significant when the *p*-value was <0.05. The statistical analysis was performed using GraphPad Prism (8.0.1), and the graphs were generated with the same program.

### 2.3. RNA Extraction and Sequencing

RNA sequencing (RNA-seq) was performed on lysed frozen PBMCs from 48 randomly selected intervention study participants (19 in the placebo group, 10 in the low dose group, and 19 in the high dose group). These individuals were the same as those subjected to *ex vivo* PBMC analysis. Each participant had paired samples taken at baseline and on day 14 of the research prior to infection with diarrheagenic *E. coli*, totaling 96 samples. On the study day, the PBMCs were lysed using buffer RLT (Qiagen 79216, Germantown, MD, USA) and stored frozen until the RNA extraction day. On that day, the samples were thawed, and the total RNA from the cells was extracted according to the manufacturer’s procedure using the RNeasy mini kit (Qiagen 74106, Germantown, MD, USA). Following that, Implen NanoPhotometer N60/N50 was used to quantify the extracted RNA, and the quality and integrity of the RNA samples were checked using the Agilent 2200 TapeStation system (Agilent, Santa Clara, CA, USA) according to the manufacturer’s protocol. RNA samples with an RNA integrity number (RIN) of 8 were used for library preparation. Library construction and sequencing were performed by Novogene (Milton Road, Cambridge, UK), where 2 × 150 bp RNA-seq reads were obtained using Illumina sequencing using a strand-aware library preparation technique.

### 2.4. Differential Gene Expression Analysis (DGE)

The sequence data were used to perform differential gene expression analysis (DGE) at NIZO (NIZO, Ede, The Netherlands) using a custom bioinformatics pipeline. First, quality control of the raw sequencing data was performed using Fast QC (v.0.11.9) and MultiQC (v.1.9). Reads were then pre-processed using fastp (v.0.20.0) using a sliding window quality score-based trimming (window width 4 bases, minimum window Q score 15). Reads less than 60 bp long after quality trimming were removed. Surviving high-quality reads were pseudo-aligned with Kallisto (v.0.46.10) to the human transcriptome (Ensemble release 101, GRCh38), taking into account how stranded the library was (e.g., using the “–rf-stranded” flag) and using 50 bootstraps and the GC bias correction (“–bias” flag). For diagnostic purposes, reads from a subset of samples were aligned to the human genome (Ensemble release 101, GRCh38) using STAR (v.2.7.5a). The resulting alignments were imported to SeqMonk (v.1.47.1) to obtain an overview of the proportion of reads aligning to introns, exons, mtDNA, and rRNA.

DGE analysis was performed using custom scripts written in R (v.4.0.2). The biomaRt (v.2.46.0) package was used to obtain and match gene and transcript identifiers (using the 101 release of Ensembl, the same as the transcriptome version used as a reference in the pseudoalignment). The tximport (v.1.18.0) package was used to import data from Kallisto output files into R (v. 4.0.2).

The DGE analysis was implemented as 3 different workflows based on 3 different R packages for DGE: DESeq2 (v.1.30.0), edgeR (v.3.32.0), and sleuth (v.0.30). DESeq2 and edgeR aggregate transcript counts to gene-level before performing per-gene statistical tests. Sleuth performs per-transcript statistical tests before aggregating the resulting *p*-values to gene-level. In all 3 flows, multiple comparison adjustment was performed on *p*-values using the Benjamini–Hochberg FDR procedure. Adjusted *p*-values smaller than 0.05 were considered significant. All 3 DGE workflows rely on likelihood ratio tests (LRTs) comparing the goodness of fit of a “full” model against that of a “reduced” model, where the full model contains the factor of interest while the reduced model does not. To exemplify, for the main outcome of interest in this analysis (i.e., group-specific changes in time, showing differential trends between the placebo group and the high dose group), the full model was “~time + group + group: time” while the reduced model was “~time + group”. Thus, the full model included the interaction between the fixed effects of “time” and “group” (group: time), while the reduced model did not. For the secondary analyses focusing on the effect of time on gene expression (i.e., non-group-specific effect), the full model was “~subject + time” while the reduced model was “~subject”.

The results of all 3 DGE workflows were integrated and compared. Genes found to be significant by all 3 workflows were regarded as being differentially expressed with very high confidence, while genes found by only one workflow were regarded as likely spurious. Results were visually summarized using Venn and Euler diagrams to assess the degree of consensus in the results of the 3 workflows. The tidyverse (v.1.30) package was used for data wrangling. The ggplot2 (v.3.3.2) and ComplexHeatmap (v.2.6.2) packages were used for visualizations.

## 3. Results

### 3.1. Baseline Characteristics of the Participants

In the GIGA study [26], a total of 120 healthy male volunteers were stratified and randomized into one of the three different study groups: placebo, low dose WPC, and high dose WPC. The current study used a randomly selected sample of 48 out of the 120 participants in the GIGA trial. The age and BMI of research participants did not differ statistically between the groups. Table 2 summarizes the baseline characteristics of these 48 subjects.

### 3.2. Ex Vivo Stimulation of Monocytes, mDC, and pDC by Diarrheagenic E. coli and TLR Ligands

To study the effects of WPC consumption on the innate immune cell response, PBMCs were isolated from freshly drawn blood samples on day 1 (baseline) and on day 14, and were stimulated with either fixed whole diarrheagenic *E. coli* strain E1392/75-2A, LPS (TLR4 ligand), flagellin (TLR5 ligand), or with RPMI medium as the negative control. Flow cytometry analysis was used to measure intracellular IL-6 and TNF-α production in (classical) monocytes, mDCs, and pDCs (Supplementary Figure S1). The results are given as a percentage of all viable monocytes or mDCs that were double-positive (producing both cytokines), or as a percentage of all IL-6 producing cells (any IL-6 positive), or as a percentage of all TNF-α producing cells (any TNF-α positive). The responses of the three study groups were compared before and after two weeks of dietary intervention.

**Figure 2:**
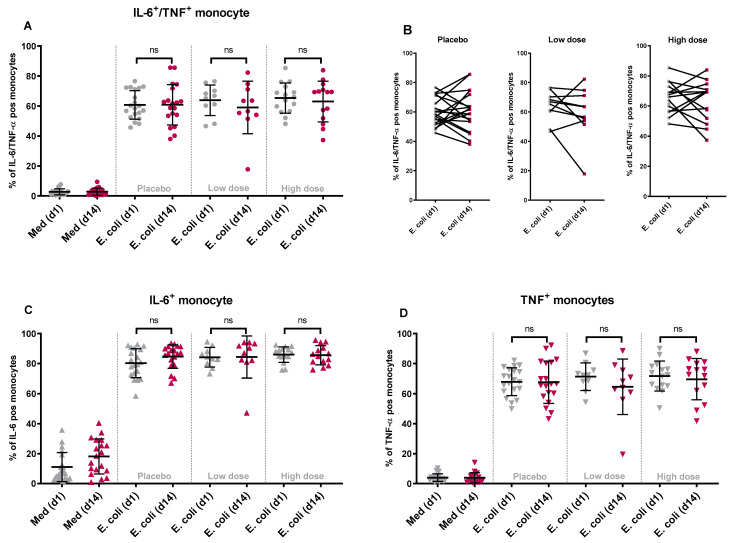
The changes in the percentage of double-positive and single-positive monocytes upon stimulation with diarrheagenic *E. coli*: following product consumption for two weeks, the percentage of monocytes producing IL-6 and TNF-α simultaneously did change significantly after an *ex vivo* stimulation with diarrheagenic *E. coli* (1E7 CFU) when comparing WPC high dose with placebo or WPC low dose group (**A**). The participants’ individual responses between day 1 and day 14 after stimulation with *E. coli* are shown in different dose groups (**B**). Similarly, when comparing the percentage of any IL-6 (**C**) or any TNF-α (**D**) producing cells, no significant variation in the responses between WPC high dose and placebo or WPC low dose was noted. (ns = non-significant *p* value).

**Figure 3:**
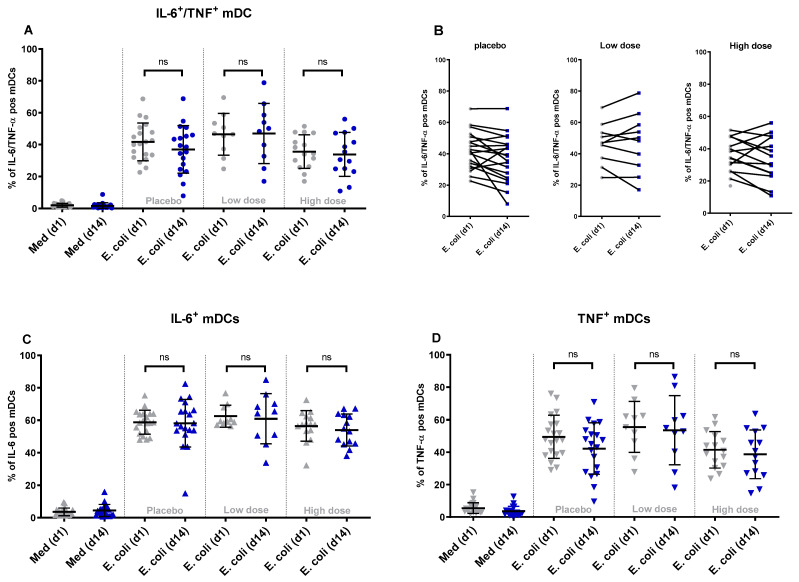
The changes in the percentage of double-positive and single-positive mDCs upon stimulation with diarrheagenic *E. coli*: after two weeks of product consumption, the percentage of mDCs producing IL-6 and TNF-α simultaneously did not show any significant change after *ex vivo* stimulation with diarrheagenic *E. coli* (1E7 CFU) in any of the WPC dose groups (**A**). The participants’ individual responses between day 1 and day 14 after stimulation with E. coli are shown in different dose groups (**B**). Looking at the % of any IL-6 producing (**C**) or any TNF-α producing mDCs (**D**), no significant difference between WPC dose groups was concluded. (ns = non-significant *p* value).

In all three groups, diarrheagenic *E. coli* stimulation of PBMCs isolated on the baseline and day 14 resulted in a comparable percentage of IL-6 and TNF-α positive (double-positive) monocytes (Figure 2A) and mDCs (Figure 3A). When comparing individual donors’ responses (% of double-positive cells) to *E. coli* stimulation at the baseline and day 14, individual responses varied between donors. This observation holds true for both monocytes (Figure 2B) and mDCs (Figure 3B). Similar results were obtained for any IL-6 positive and any TNF-α positive monocytes and mDCs before and after product consumption. The findings indicate no significant differences in the percentage of any IL-6 or TNF-α positive monocytes (Figure 2C,D) or any IL-6 or TNF-α positive mDCs (Figure 3C,D) following diarrheagenic *E. coli* stimulation in any of the study groups.

As with *E. coli*, stimulation of PBMCs with TLR ligands did not increase cytokine responses in the intervention groups. Although some donors displayed different responses between baseline and day 14, these changes were not consistent across groups and cell types (Supplementary Figure S2A–F) and occurred primarily in the placebo group. Moreover, the stimulation of PBMCs did not significantly affect the percentage of IL-6 and TNF-α producing pDCs evaluated on the baseline compared to day 14 (Supplementary Figure S2G–I), although it should be mentioned that responses in pDCs to the stimuli utilized were very low. Overall, these results indicate that the nutritional intervention with WPC did not modify the responses of (classical) monocyte, mDC, or pDC to *ex vivo* stimulation.

### 3.3. Gene Expression Analysis

In addition to *ex vivo* stimulation of PBMCs, mRNA was isolated from these cells at both time points and was sequenced to analyze the gene expression. Changes in gene expression patterns of PBMC from all three study groups were investigated with two goals in mind: to identify the impacts of the dietary intervention (group-specific differential gene expression (DGE)) and to detect changes over time irrespective of the dietary intervention (baseline vs. day 14).

**Figure 4:**
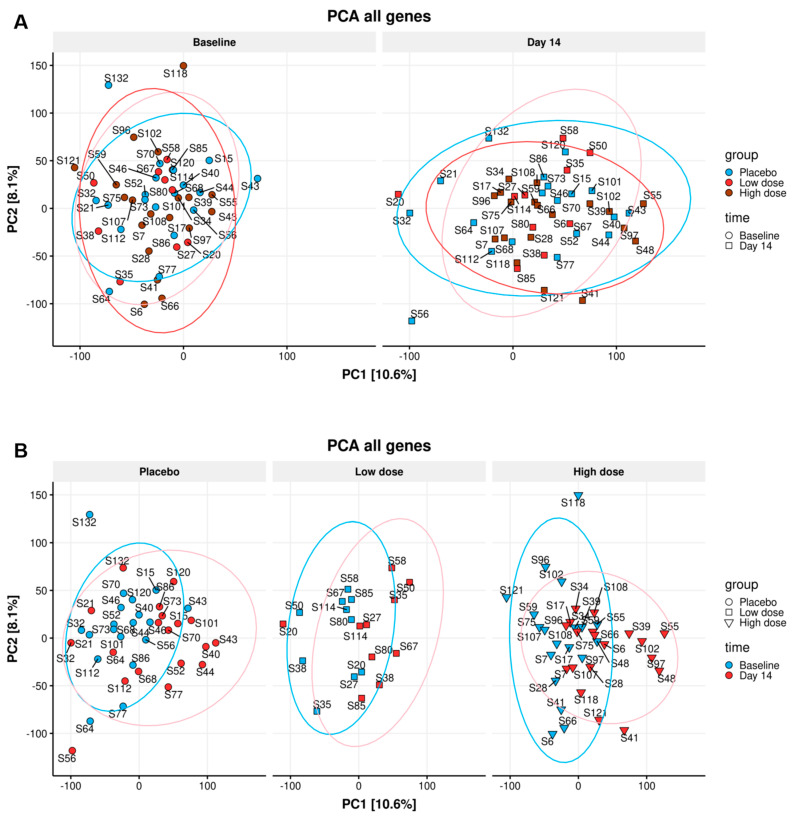
Principle component analysis (PCA) for DGE: The differences in the DGE of all individuals on the baseline and all individuals on day 14 (**A**) of placebo, WPC low dose, and WPC high dose, no statistically significant gene expression difference in three dose groups was identified (**A**). Additionally, the gene expression pattern of all participants in two time points did not lead to a clear difference (**B**).

An exploratory data analysis of the sequenced mRNA samples was initially performed on the gene counts obtained from the pseudoalignment to detect any potential outliers and discern global trends in the data. To investigate and visualize the variation and patterns of our dataset in a single figure, results obtained from the various immunological analyses were used to conduct a two-dimensional principal component analysis (PCA). The PCA indicates the amount of variation retained by each principal component (PC1 scores on the *x*-axis and PC2 scores on the *y*-axis). For the differences in the DGE, the amount of variation explained by PC1 and PC2 is 10.6% and 8.1%, respectively. No differences were observed on the PCA plots between any of the study groups at day 14 (Figure 4A), and apparent differences between baseline and day 14 were seen in all study groups (Figure 4B). This indicates a time-dependent difference in PBMCs gene expression in all groups that was not linked to the nutritional intervention since the samples from the placebo group showed the same trend.

### 3.4. Differential Gene Expression (DGE)

**Figure 5:**
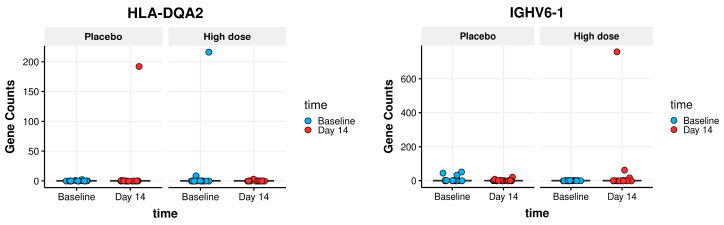
Outlier-driven spurious findings of group-specific DGE genes: Boxplots showing outlier-driven spurious findings of two group-specific DGE genes. The boxplot itself (showing the interquartile range of each group) is compressed to the line by the extreme amplitude of a few outlier values: one outlier in the high dose group for the IGHV6-1 gene; two outliers for the HLA-DQA2 gene: one in the placebo group and one in the high dose group.

The PBMCs’ gene expression was studied to identify any group-specific changes in gene expression after consuming the study product. All samples had more than 30 million raw sequencing read pairs (range 30–38 million read pairs). Around 26,000 genes had enough counts to be assessed using DGE analysis. The analysis focusing on the primary goal of identifying group-specific DGE, did not find any genes that were (significantly) differentially expressed in any of the three groups at day 14. These results indicate no effects of nutritional intervention on gene expression patterns. No genes were found to display group-specific DGE by either sleuth- or DESeq2- analysis, and only three genes were found to be differentially expressed by edgeR analysis. At a closer inspection, in all three genes, the effect was found to be spurious, outlier-driven (two of them are shown in Figure 5).

**Figure 6:**
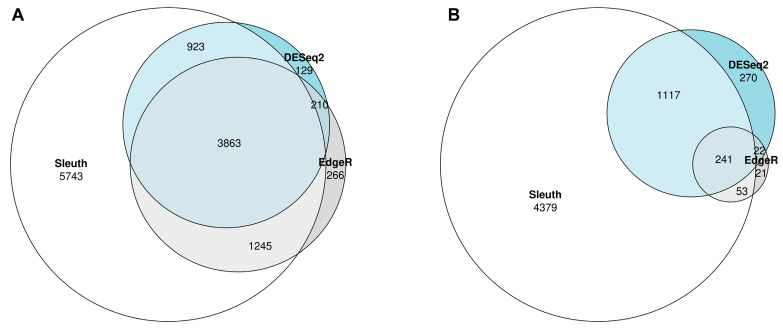
Euler diagram of the DGE results from the three different workflows: Three different workflows, namely Sleuth, DESeq2, and EdgeR were applied to look at changes in PBMCs gene expression over time (baseline vs. day 14) in the participants of the high dose group (**A**) and the placebo group (**B**). In the high dose group, 3863 genes were differentially expressed by all three workflows (**A**). The number of differentially expressed genes in the placebo was 241 when looking at the genes found by all three workflows.

As a secondary goal, DGE analyses were also performed to identify genes that changed expression between baseline and day 14 of the study. Two analyses were performed, one focusing only on high dose group participants and the other focusing on the placebo group. The analyses found 3863 genes that were differentially expressed over time in the high dose group (Figure 6A) and 241 genes in the placebo group (Figure 6B). These numbers refer to high confidence genes found to be significant by the consensus of all three DGE flows: sleuth, DESeq2, and edgeR.

This indicates that even though no group-specific changes in gene expression were noted, in the high dose group compared to the placebo group, more genes were significantly changed in time. The list of (consensus) differentially expressed genes (in time) in the high dose group was extracted to investigate why this is the case. Then all the differentially expressed genes in the placebo group were removed from the list, even if non-consensus (e.g., even if found in just one of the three DGE workflows). The remaining 650 genes in the list were examined. In all cases, these genes showed the same trend in the placebo group as in the high dose group, without reaching statistical significance (Four selected genes are shown in Supplementary Figure S2A–D as examples). Overall, this means that, although a higher number of genes were differentially expressed in the high dose group, the differences were not significant and are not linked to the ingestion of the study product.

## 4. Discussion

The present study shows that a two-week dietary intervention with a whey protein concentrate (WPC) compared to placebo had no effect on the myeloid innate immune cells response to TLR ligand-induced activation and did not induce differential gene expression patterns in PBMCs of the study participants.

We recently demonstrated that a primary *in vivo* challenge of healthy volunteers with diarrheagenic *E. coli* bacteria strain E1392/75-2A at doses as low as 1E6 CFU resulted in protection against secondary infection three weeks later [27]. The primary infection primed monocytes and mDCs for an increased IL-6 and TNF-α production after restimulation with *E. coli* or TLR ligands *ex vivo*. This enhanced innate immune response could be due to *in vivo* innate immune training.

Although innate immune training has been extensively studied *in vitro* and several food components have been shown to induce trained immunity, little evidence exists for trained innate immunity *in vivo* after nutritional intervention. The *in vitro* training of monocytes has been demonstrated using raw milk, bovine milk IgG (bIgG) as a purified molecule, or whey protein preparations, including WPC [19]. Interestingly, the depletion of bIgG from the whey protein extracts did not entirely eliminate the training effect, indicating the presence of (an)other active ingredient(s) in the whey preparation. The current study product is whey protein concentrate (WPC) from raw bovine milk, the same product applied in the previously mentioned *in vitro* study [19]. WPC is rich in bioactive components, including whey proteins (e.g., immunoglobulins, Milk Fat Globule Membrane proteins) and phospholipids. It was processed mildly enough to ensure that bIgG and other proteins were not denatured (based on internal validation). Therefore, we investigated whether the *in vitro* training results can be translated into *in vivo* immune effects on monocytes after WPC oral ingestion.

Prior to and after a two-week nutritional intervention in healthy adult male volunteers, PBMCs were isolated and stimulated with diarrheagenic *E. coli* bacteria as well as with TLR ligands. No changes in the cytokine response of (classical) monocytes, mDCs, and pDCs were seen between the study groups, which indicates no training effects *ex vivo*. Nevertheless, we must keep in mind that the study participants were healthy male adults (18–55 years) with no known medical conditions based on the study inclusion criteria. Arts et al., similar to our findings, were unable to replicate the *in vitro* results for trained immunity induced by BCG vaccination *in vivo* [28], and oral ingestion of β-glucan did not enhance the innate immune response in humans [29]. These studies were performed in a comparable group of people comprised of healthy adults ranging between 20 and 34 years old. However, the immune system competence is different in infants, the elderly, and immunocompromised individuals. TLR function, for example, is impaired in the context of aging, resulting in a decreased innate immune response and increased susceptibility to bacterial and viral infections [30]. In elderly women, it was demonstrated that ingestion of bovine lactoferrin (bLF) could partially restore TLR7/8 responsiveness in pDC [31]. Therefore, even though we could not demonstrate the effect of WPC on innate immune responses in healthy male adults, it is possible that elderly subjects might benefit from the dietary intervention, although future studies are required to substantiate this notion.

As it is well established that dietary components can influence cells’ gene expression locally or systemically [32,33], we also evaluated the effect of the nutritional intervention on gene expression in PBMCs of the participants. The intervention did not induce differential group-specific gene expression patterns of the PBMCs of the volunteers in any of the study groups. As a result, no downstream analyses could be performed (e.g., gene set enrichment analysis or Gene Ontology enrichment analysis). The same line of reasoning discussed above can also explain the gene expression findings. The gene expression regulatory machinery in healthy young individuals is sufficiently competent to maintain homeostasis. This is not entirely true in immunocompromised individuals, who most likely may benefit from nutritional intervention. However, further research in specific populations is needed to validate this.

Interestingly, a time effect on gene expression patterns was noted in all groups regardless of the study product. Between baseline and day 14, large-scale changes in gene expression were seen in all study participants. These alterations occurred regardless of the study group and were observed in both the placebo and high dose groups. Further in-depth analysis of these findings revealed no statistically significant difference, and the observed trend was comparable in the placebo and high dose groups. A possible explanation for the time effect observed is that the study’s dietary restriction guidelines resulted in these changes in gene expression. All study participants followed identical guidelines that began concurrently with the intervention study and resulted in considerable changes in dietary habits and may explain the findings.

The data presented here in a subgroup of participants of the GIGA study reflects the findings on the clinical effect of WPC on diarrheagenic *E. coli* infection symptom score and microbiota composition [26]. After the two-week dietary intervention, participants of the GIGA study were orally inoculated with 1E10 CFU of the live-attenuated diarrheagenic *E. coli* strain E1392/75-2A. On days 11–18 and two weeks later, on day 28, the progression of clinical diarrheal symptoms was evaluated. The clinical outcomes regarding diarrhea incidence, stool frequency, and gastrointestinal discomfort (Gastrointestinal Rating Score, GSRS) were comparable in all three study groups and could not demonstrate an effect of the nutritional intervention. Although participants experienced clinically mild diarrhea following the oral challenge, no dose group influence on diarrhea outcomes was observed. Finally, when the intervention effects on microbiota composition were studied, a similar time-dependent effect on microbiota was noted across all study groups. These findings corroborate our results on gene expression and *ex vivo* PBMC stimulation in a subset of the study population.

## 5. Conclusions

In conclusion, in the current study, we could not demonstrate an effect of WPC ingestion on either myeloid cells’ responsiveness to TLR agonists or the gene expression pattern of PBMCs.

It is important to point out that this study was a post-hoc study. The original study was performed on 120 volunteers who had the dietary intervention followed by a challenge with mild diarrhea-inducing *E. coli* [26]. In this post-hoc analysis, we focused on the direct effects of WPC ingestion on immune function and gene expression rather than on the resistance to infection as assessed by the *E. coli* challenge. The study was performed on a randomized subsample of the original study and should thus be considered as a pilot study rather than a fully powered analysis. However, given the lack of any significant trends between the placebo and active group, we do not think that the low number of participants has masked the outcomes of this study. If the work presented here is followed up, a larger group of participants and a longer study duration will be needed, as a two-week intervention study is too short to consider indirect microbiota-induced effects. An earlier start of the dietary restrictions before the start of the nutritional intervention is also recommended, as these may have induced changes in the microbiome before the start of the intervention itself [26].

Finally, performing the study in a more vulnerable group (e.g., with decreased TLR responsiveness) could potentially increase the chance of detecting changes in immune function and gene expression in PBMCs.

## Figures and Tables

**Table 1:** Antibodies panel used for PBMCs immunological assay.

Antibody	Panel	Fluorochrome	Host	Isotype	Light Chain	Clone	Company	Catalog Number
α-CD3	1	FITC	mouse	IgG1	κ	UCHT1	Biolegend	300406
α-CD11c	1	BV 421	mouse	IgG1	κ	3.9	Biolegend	301628
α-CD14	1	Percp-Cy5.5	mouse	IgG1	κ	HCD14	Biolegend	325622
α-CD19	1	FITC	mouse	IgG1	κ	SJ25C1	Biolegend	363008
α-CD20	1	FITC	mouse	IgG2b	κ	2H7	Biolegend	302304
α-CD56	1	FITC	mouse	IgG1	κ	HCD56	Biolegend	318304
α-HLA-DR	1	BV 510	mouse	IgG2a	κ	L243	Biolegend	307646
α-CD123	1	PE-Cy7	mouse	IgG1	κ	6H6	Biolegend	306010
α-IL-6	2	PE	rat	IgG1	κ	MQ2-13A5	Biolegend	501107
α-TNF-α	2	AF647	mouse	IgG1	κ	MAb11	Biolegend	502916

Company affiliation is Biolegend (San Diego, CA, USA).

**Table 2:** Baseline characterization of the study participants.

Variable		Placebo	WPC Low Dose	WPC High Dose
Number		19	10	19
Age	Mean (SD)	36.29 (11.6)	34.16 (11.93)	33.7 (9.98)
BMI (kg/m^2^)	Mean (SD)	24.43 (2.24)	23.85 (2.93)	24.02 (2.81)

## Data Availability

The data presented in this study are available on request from the corresponding author.

## Supplementary Materials

The following supporting information can be downloaded at: https://www.mdpi.com/article/10.3390/nu16050592/s1, Figure S1: Gating strategy for monocytes, mDCs, and pDCs phenotyping: PBMCs were selected in the FSC/SSC plot, and the duplets were gated out. HLA-DR^+^CD14^+^ cells were selected as the monocytes, and dead cells were removed. CD3^+^, CD19^+^, CD20^+^, CD56^+^ and dead cells from the HLA-DR^+^CD14^−^ population were excluded. From there, CD11c^+^ cells were considered as mDCs, and CD123^+^ cells were named pDCs. Within monocyte, mDC, and pDC populations, the percentage of cells that were producing IL-6, TNF-α or both cytokines were determined. Figure S2. The changes in the % of double-positive monocytes, mDCs, and pDCs following the stimulation with TLR ligands and *E. coli*: The percentage of monocytes (A–C), mDCs (D–F), and pDCs (G–I) simultaneously producing both IL-6 and TNF-α following the stimulation with LPS, flagellin, and whole *E. coli* bacteria were quantified. The comparison was made between each dose group’s baseline and day 14 responses within each TLR stimulation. Figure S3: An example of genes showing the same trend in both Placebo and High Dose groups: The selected genes PSMA4 (A), CCDC191 (B), UBE2V2 (C), and SLIRP (D) show the same trend in both placebo and high dose groups yet not reaching statistical significance. Figure S4. MA plot showing the top 50 most differentially expressed genes in time in high dose group subjects (as determined by DESeq2-derived *p*-value): X-axis shows the mean expression levels of the genes; y-axis shows the fold-change in gene expression from Baseline to Day 14.
