# Supplementary material for: Dietary Intervention with Whey Protein Concentrate Does Not Affect Toll-like Receptor Responses and Gene Expression Patterns in Peripheral Blood Mononuclear Cells of Healthy Volunteers
Source: Nutrients. 2024 Feb 22;16(5):592. doi: 10.3390/nu16050592 (PMC10934438; doi:10.3390/nu16050592)
Supplement: Supplementary file 1 [file nutrients-16-00592-s001.zip › nutrients-2862487-supplementary.pdf]

Supplementary data

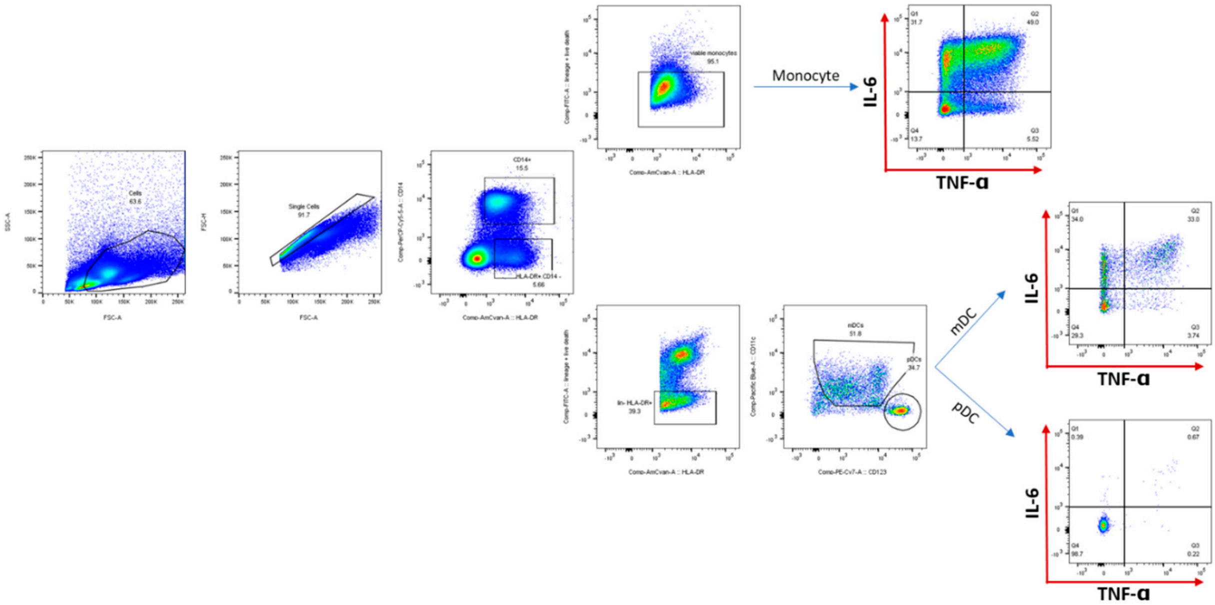

Figure S1. Gating strategy for monocytes, mDCs, and pDCs phenotyping: PBMCs were selected in the FSC/SSC plot, and the duplets were gated out. HLA-DR^{+}CD14^{+} cells were selected as the monocytes, and dead cells were removed. CD3^{+}, CD19^{+},CD20^{+}, CD56^{+} and dead cells from the HLA-DR^{+}CD14^{-} population were excluded. From there, CD11c^{+} cells were considered as mDCs, and CD123^{+} cells were named pDCs. Within monocyte, mDC, and pDC populations, the percentage of cells that were producing IL-6, TNF-α or both cytokines were determined.

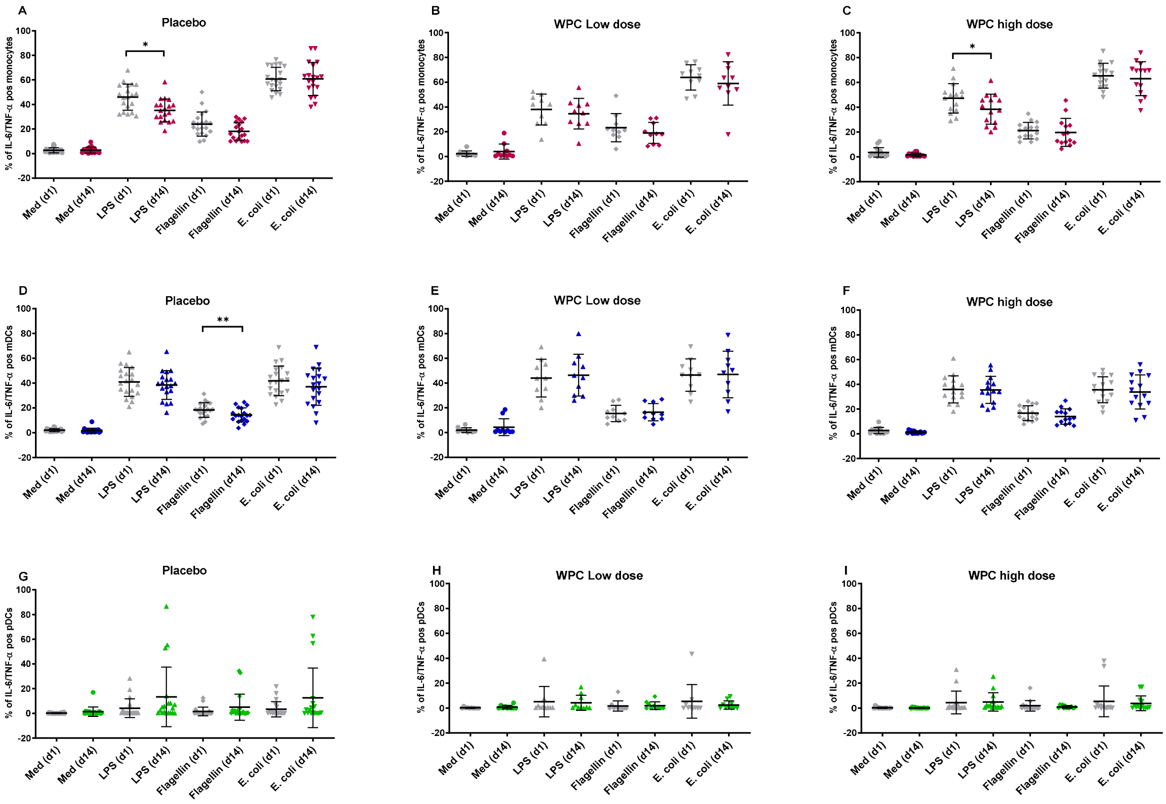

Figure S2. The changes in the % of double-positive monocytes, mDCs, and pDCs following the stimulation with TLR ligands and *E. coli*: The percentage of monocytes (A–C), mDCs (D–F), and pDCs (G–I) simultaneously producing both IL-6 and TNF-α following the stimulation with LPS, flagellin, and whole *E. coli* bacteria were quantified. The comparison was made between each dose group's baseline and day 14 responses within each TLR stimulation.

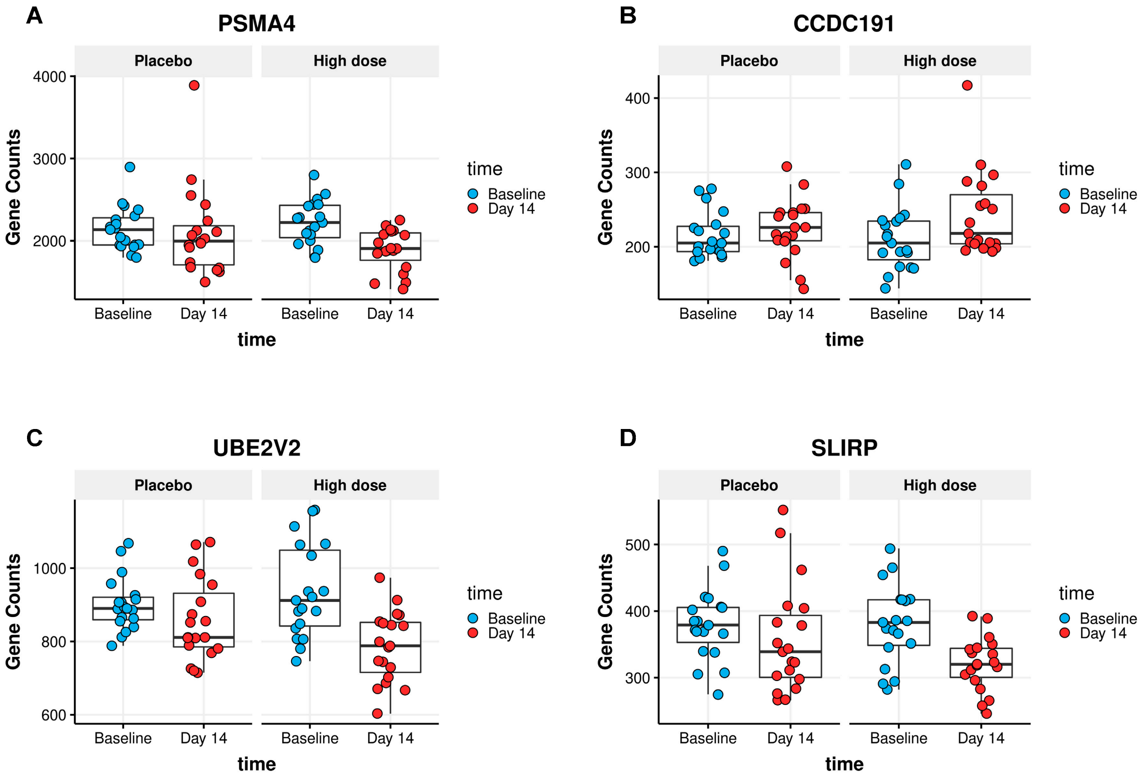

Figure S3. An example of a gene showing the same trend in both Placebo and High Dose groups: yet not reaching statistical significance in the Placebo group.

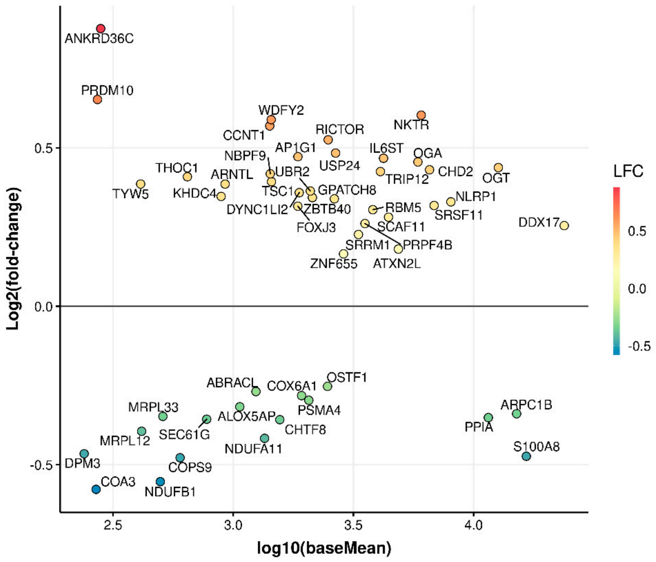

Figure S4. MA plot showing the top 50 most differentially expressed genes in time in high dose group subjects (as determined by DESeq2-derived p-value): X-axis shows the mean expression levels of the genes; y-axis shows the fold-change in gene expression from Baseline to Day 14.

Study Inclusion criteria

In order to be eligible to participate in this study, a subject must meet all of the following criteria:

Substantial:

1. Male
2. Age between 18 and 55 years.
3. BMI ≥18.5 and ≤30.0 kg/m^2.
4. Healthy as assessed by the NIZO health questionnaire.

Procedural:

5. Ability to follow Dutch verbal and written instructions.
6. Availability of internet connection.
7. Signed informed consent.
8. Willing to accept disclosure of the financial benefit of participation in the study to the authorities concerned.
9. Willing to accept use of all encoded data, including publication, and the confidential use and storage of all data for at least 15 years.
10. Willing to comply with study procedures, including collection of stool and blood samples.
11. Willingness to abstain from high calcium containing products during the study.
12. Willingness to abstain from alcoholic beverages three days before, during and for 4 days after diarrheagenic *E. coli* challenge.
13. Willingness to abstain from medications that contain acetaminophen, aspirin, ibuprofen, and other nonsteroidal anti-inflammatory drugs, (OTC) antacids and antimotility agents (eg, loperamide) on the three days before, during and for 4 days after diarrheagenic *E. coli* challenge.
14. Willingness to abstain from probiotics and prebiotics/fibers starting from run-in and during the entire study.
15. Willingness to give up blood donation starting at run-in and during the entire study.

Study Exclusion criteria

A potential subject who meets any of the following criteria will be excluded from participation in this study:

Substantial:

1. Acute gastroenteritis in the 2 months prior to inclusion.
2. Any confirmed or suspected immunosuppressive or immunodeficient condition including human immunodeficiency virus infection (HIV).
3. Disease of the GI tract, liver, bile bladder, kidney, thyroid gland (self-reported), except for appendicitis.
4. History of microbiologically confirmed ETEC or cholera infection within 3 years prior to inclusion.
5. Symptoms consistent with Travelers' Diarrhea concurrent with travel to countries where ETEC infection is endemic (most of the developing world) within 3 years prior to inclusion, OR planned travel to endemic countries during the length of the study.
6. Vaccination for, or ingestion of cholera within 3 years prior to inclusion, including studies at NIZO.
7. Occupation involving handling of ETEC or *Vibrio cholerae* currently, or within 3 years prior to inclusion.
8. Vaccination for, or ingestion of ETEC or *E coli* heat labile toxin, including *E. coli* challenge studies at NIZO.
9. Evidence of current excessive alcohol consumption (>4 consumptions/day or >20 consumptions/week) or drug (ab)use, and not willing/able to stop this during the study.
10. Known allergy to the following antibiotics: ciprofloxacin, trimethoprim, sulfamethoxazole, and penicillins.
11. Reported average stool frequency of >3 per day or <1 per 2 days.
12. Use of antibiotics (up till 6 months prior to inclusion), norit, laxatives, cholestyramine, antacids H2 receptor antagonists or proton pump inhibitors (during 3 months prior to inclusion).
13. Use of immunosuppressive drugs (e.g., cyclosporine, azathioprine, systemic corticosteroids, antibodies).
14. Vegans.
15. Mental status that is incompatible with the proper conduct of the study.
16. A self-reported milk allergy, lactose intolerance or sensitivity to dairy ingredients.

17. Not having a general practitioner, not allowing disclosure of participation to the general practitioner or not allow to inform the general practitioner about abnormal results.

18. Participation in any clinical trial including blood sampling and/or administration of substances starting 1 month prior to study start and during the entire study.

19. Personnel of NIZO or FrieslandCampina, their partner and their first and second degree relatives.

Flow cytometry staining protocol

1. Harvest the cells from the culture plate and transfer the entire content of the wells (˜1 mL) into a 1mL deep well NUNC plate.
2. Spin down the cells for 3 min 400 g and discard the supernatant by flipping the plates and discarding the medium in the sink.
3. Resuspend the cells in 200 µL of FACS buffer and transfer them to a standard (500 µL) NUNC plate (˜2 × 10^6/well).
4. Spin down for 3 min at 400 g and discard the supernatant.
5. Add 50 µL of (extracellular markers antibody mix + Fc Block) diluted in FACS buffer.
6. Wrap in aluminium foil and incubate on ice for 30 min.
7. Add 200 µL FACS buffer.
8. Spin down for 3 min 400 g and discard the supernatant.
9. Wash with 200 µL of PBS 2 times (2X) and discard the supernatant after each wash.
10. Stain cells with 50 µL of freshly-made FVD520 (diluted 400X) in PBS.
11. Wrap in aluminium foil and incubate in the fridge for 20 min (cold and dark).
12. Add 200 µL FACS buffer.
13. Spin down for 3 min at 400 g and discard the supernatant.
14. Resuspend in 100 µL IC Fix buffer.
15. Incubate 45 min RT.
16. Add 100 µL of Perm buffer.
17. Spin down for 3 min at 400 g and discard the supernatant.
18. Wash cells twice (2X) with 200 µL Perm buffer (spin down 3 min at 400 g) and discard the supernatant after each wash.
19. Resuspend in 50 µL intracellular antibody mix in perm buffer.
20. Wrap in aluminum foil and incubate in the fridge for 20 min (cold and dark).
21. Add 200 µL Perm buffer.
22. Spin down for 3 min 400 g and discard the supernatant.
23. Wash with 200 µL of FACS buffer 2 times (2X) and discard the supernatant after each wash.
24. Resuspend pellet in 300 µL FACS buffer and measure on the flow cytometer.
